# Full-fat soybean origin and thermal processing affect protein secondary structure, in vitro digestion kinetics, and predicted standardized ileal amino acid digestibility in broiler chickens

**DOI:** 10.1186/s40104-026-01512-w

**Published:** 2026-11-02

**Authors:** Felix M. Njeri, Adriana Leticia Ciola, Lonita A. Manoa, Munene Kithama, Joyce G. Maina, Charles K. Gachuiri, Lee-Anne Huber, Elijah G. Kiarie

**Affiliations:** 1 Department of Animal Biosciences, University of Guelph, Guelph, ON Canada; 2 Department of Animal Sciences, Pwani University, Kilifi, Kenya; 3 Department of Animal Production, University of Nairobi, Nairobi, Kenya

**Keywords:** ATR-FTIR spectroscopy, Full-fat soybean, In vitro digestion, Protein secondary structure, Thermal processing

## Abstract

**Background:**

This study evaluated the chemical composition, protein secondary structure, crude protein (CP) solubilization, and in-vitro amino acid (AA) digestibility of three Kenyan full-fat soybean (FFSB) samples (S1, S2, S3) with unknown processing histories and compared with Canadian samples: raw FFSB, extruded FFSB at 160.0 °C, and FFSB roasted at 136.7 °C, 151.7 °C, or 154.4 °C. Protein structure was characterized using attenuated total reflectance-Fourier transform infrared spectroscopy. A two-phase in-vitro digestion model was used to determine CP solubilization and AA digestibility. Observed in-vivo standardized ileal digestibility (SID) values from a study using FFSB roasted at 136.7 °C were regressed on corresponding in-vitro AA digestibility values to generate predicted SID values.

**Results:**

Thermal processing reduced trypsin inhibitor activity (TIA) in Canadian FFSB from 17.42 TIU/mg in raw FFSB to 3.85 TIU/mg in extruded FFSB and to 4.77, 4.59, and 2.30 TIU/mg in samples roasted at 136.7, 151.7, and 154.4 °C, respectively. In contrast, Kenyan Samples 1, 2, and 3 had residual TIA values of 8.00, 6.00, and 12.00 TIU/mg, respectively. Protein secondary structure differed among samples (*P* < 0.001), with the 136.7 °C-roasted sample having greater aggregated β-sheet and β-turn contents and lower random coil content than most samples. CP solubilization differed among samples at each digestion time point (*P* < 0.001). At the end of the intestinal phase, processed Canadian samples had greater CP solubilization than raw FFSB but did not differ from one another, whereas Kenyan Samples 2 and 3 were comparable to Canadian raw FFSB. In-vitro AA digestibility differed among samples for all AA (*P* ≤ 0.001). For mean total AA digestibility, the 154.4 °C and 151.7 °C-roasted sample had the highest digestibility; Kenyan Sample 1 was greater than Samples 2 and 3, and Sample 3 did not differ from raw FFSB. The regression model showed agreement at the mean level but larger prediction errors for lysine, aspartic acid, and proline, indicating AA-specific limitations.

**Conclusions:**

Overall, processing conditions and ingredient origin influenced FFSB quality, with roasting at 151.7–154.4 °C producing favorable structural and digestibility responses. The variability observed among Kenyan samples highlights the need for routine quality assessment of commercial FFSB before feed formulation.

**Supplementary Information:**

The online version contains supplementary material available at https://doi.org/10.1186/s40104-026-01512-w.

## Background

Soybean remains one of the most widely utilized plant protein source in monogastric animal nutrition due to its high crude protein content and superior amino acid (AA) profile compared with other plant proteins. Full-fat soybeans (FFSB) offer both energy and protein in a single ingredient and are often used in poultry and swine diets [1]. However, the nutritional quality of FFSB is highly variable, influenced by both soybean variety and processing conditions [2]. Inadequate heat treatment can leave residual antinutritional factors (ANF) such as trypsin inhibitors and lectins, while overprocessing may induce protein denaturation and aggregation, reducing amino acid digestibility [3]. Consequently, understanding the structural and compositional variability among commercially sourced FFSB is critical for optimizing use in feed formulation.

Several processing techniques are commonly used to improve the nutritional value of FFSB, including roasting, extrusion, micronization, and jet-sploding [1, 4, 5]. Roasting typically involves dry heating at high temperatures for short durations, which can affect protein conformation in varying ways depending on temperature uniformity and exposure time [2]. In many regions, on‑farm roasting of soybeans is a common practice [6], particularly in parts of Canada and the United States, where producers roast their own soybeans to meet on‑farm feed ingredient needs [6]. While convenient, this practice introduces considerable variability due to differences in equipment, operator experience, and process control, creating challenges for nutritionists who must formulate diets with consistent nutritional value [6]. Extrusion applies high temperature, pressure, and shear force, often resulting in more complete inactivation of ANF but also promoting Maillard reactions and aggregation of protein molecules [1]. These methods yield FFSB with distinct physical and structural characteristics, yet their comparative effects on protein functionality and in vitro digestibility in broiler chickens remain incompletely characterized.

Beyond compositional analysis, recent advances in protein chemistry emphasize the importance of protein secondary structure in determining digestive behavior [7]. Protein folding and aggregation influence solubility, enzyme accessibility, and ultimately nutrient release during gastrointestinal transit [8]. Fourier‑transform infrared (FTIR) spectroscopy allows non‑destructive quantification of secondary structures, including α‑helix, β‑sheet, β‑turns, and random coils, within the amide I region, offering insights into conformational differences not apparent from proximate composition alone [9, 10]. Prior studies have shown that β-sheet-rich proteins, often stabilized by intermolecular hydrogen bonds, resist enzymatic hydrolysis and may reaggregate during digestion, while α-helical structures tend to be more labile and digestible [11, 12].

In vitro digestion models provide a controlled, mechanistic approach to examine how protein structure governs solubilization and hydrolysis kinetics [13, 14]. When combined with spectral and compositional data, these models offer a systems‑level understanding of ingredient quality and processing effects. Despite growing interest in structural characterization, limited work has explored the relationship between protein conformation and digestibility in FFSB products sourced from commercial feed mills or on‑farm operations, particularly in regions where processing histories are often unknown and ingredient quality varies widely.

To address this gap, this study evaluated the effects of processing history and ingredient origin on the chemical composition, residual trypsin inhibitor activity, protein secondary structure, crude protein solubilization kinetics, and in vitro amino acid digestibility of full-fat soybean samples. Canadian FFSB samples processed under defined extrusion or roasting conditions were compared with commercially sourced Kenyan FFSB samples of unknown processing history to capture both controlled processing responses and field-relevant ingredient variability. In addition, observed in vivo standardized ileal digestibility values were regressed against corresponding in vitro amino acid digestibility values to explore the predictive value and amino acid-specific limitations of the in vitro digestion model. We hypothesized that optimal thermal processing would improve amino acid digestibility through coordinated reductions in trypsin inhibitor activity and favorable protein structural modification, whereas under-processing or unfavorable aggregation would compromise digestibility.

## Materials and methods

### Study location, chemicals and reagents

The study was conducted at the University of Guelph, ON, Canada. Pepsin (P7000; ≥ 250 U/mg solid) and pancreatin (P1750; 4 × USP specifications) were obtained from Sigma-Aldrich (St. Louis, MO, USA). Porcine bile extract (CAS 8008-63-7) was purchased from Santa Cruz Biotechnology (Dallas, TX, USA), and the Pierce™ BCA Protein Assay Kit was purchased from Thermo Fisher Scientific (Waltham, MA, USA).

### Soybean samples

Three FFSB samples were obtained from the Kenyan market between September and December 2024. Samples 1 and 2 were collected directly from commodity vendors, whereas Sample 3 was obtained through a farmer who had purchased the product from a commercial vendor and referred the research team to the source (Table 1). All Kenyan samples had unknown processing histories and were evaluated to assess variability in FFSB quality available to end users in the Kenyan market. Sample 3 was included because its use in broiler diets on the farm reportedly coincided with elevated mortality, prompting further characterization of its processing adequacy and nutritional quality. Five Canadian FFSB samples were obtained from farms that processed soybeans on-site (Table 1). The samples consisted of one raw, unprocessed FFSB sample and four processed FFSB samples. The processed samples included one extruded sample produced using an electrically powered Insta-Pro extruder at 160.0 °C for 25 s, and three roasted samples produced using an electrically powered Dilts-Wetzel roaster. The roasted samples were processed at 136.7 °C for 90 min, 151.7 °C for 90 min, and 154.4 °C for 50 min. The reported roasting temperatures represented exit temperatures.

**Table 1:** Analyzed chemical composition (dry matter basis) of the full-fat soybean samples

Item^1^	Raw	Extruded 160.0 °C	Roasted 136.7 °C	Roasted 151.7 °C	Roasted 154.4 °C	Sample 1	Sample 2	Sample 3
Dry matter, %	93.41	93.33	94.25	94.84	94.85	95.41	91.37	93.64
Crude protein, %	36.66	35.23	34.57	35.94	35.49	37.90	40.99	38.67
IDF, %	21.51	24.8	26.65	23.99	23.31	-	-	-
SDF, %	1.36	1.23	0.95	1.14	1.26	-	-	-
Acid detergent fiber, %	-	-	-	-	-	4.60	6.15	6.42
Neutral detergent fiber, %	-	-	-	-	-	8.04	10.21	9.93
Fat, %	19.20	18.79	20.45	19.24	20.26	22.02	19.04	19.08
Trypsin inhibitors, TIU/mg	17.42	3.85	4.77	4.59	2.30	8.00	6.00	12.00
Amino acids								
Arg, %	2.56	2.43	2.42	2.42	2.40	2.30	2.75	2.57
His, %	0.28	0.30	0.34	0.29	0.30	0.87	0.91	0.94
Ile, %	1.81	1.75	1.71	1.69	1.71	1.79	2.38	1.87
Leu, %	2.99	2.84	2.83	2.80	2.89	2.90	3.31	3.09
Lys, %	2.70	2.53	2.51	2.43	2.52	2.01	2.15	2.26
Met, %	0.56	0.53	0.56	0.52	0.53	0.53	0.54	0.58
Phe, %	1.84	1.78	1.77	1.75	1.83	1.84	3.00	2.02
Thr, %	1.35	1.28	1.33	1.27	1.29	1.27	1.26	1.36
Val, %	1.89	1.84	1.78	1.76	1.79	1.71	1.84	1.87
Ala, %	1.75	1.65	1.65	1.61	1.67	1.52	1.49	1.59
Asp, %	4.97	4.70	4.65	4.58	4.70	3.73	3.92	4.07
Cys, %	0.71	0.77	0.74	0.71	0.75	0.67	0.76	0.85
Glu, %	7.61	7.11	7.07	7.01	7.24	6.19	6.40	6.33
Gly, %	1.58	1.51	1.54	1.46	1.55	1.45	1.46	1.50
Pro, %	2.01	1.92	1.97	1.92	1.99	1.88	1.93	1.97
Ser, %	1.75	1.69	1.73	1.67	1.72	1.84	1.79	1.82
Tyr, %	1.12	1.03	1.06	1.03	1.08	1.58	1.63	1.57

*IDF* Insoluble dietary fiber, *SDF* Soluble dietary fiber

^1^Raw, extruded, and roasted samples represent Canadian reference full-fat soybean (FFSB) processed under defined conditions; roasting temperatures correspond to exit temperatures. Samples 1–3 were sourced from commercial vendors within the Kenyan market and represent FFSB with unknown processing histories

### Protein digestion simulation

A modified in vitro digestion protocol for broiler chickens was used, based on Ton Nu et al. [15]. In brief, from each test ingredient, 500 mg crude protein equivalents were weighed into triplicate 50-mL centrifuge tubes. Preliminary titrations were performed to establish the volumes of acid (0.1 mol/L HCl) or base (0.1 mol/L NaOH) required to achieve pH targets. For the gastric phase, 25 mL of 0.1 mol/L phosphate buffer (pH 6.0) and 2 mL of pepsin solution (27 mg/mL) were added to each sample. The pH was adjusted to 2.5 using pre-determined volumes of 1 mol/L HCl. Three randomly selected tubes were used for pH confirmation. The samples were incubated in a temperature-controlled shaker (New Brunswick™ Innova^®^ 43 Series) at 41 °C and shaking at 200 r/min for 0 and 0.5 h during the gastric phase. To simulate the intestinal phase, the gastric-digested mixtures were further mixed with 10 mL of 0.2 mol/L phosphate buffer (pH 6.8), 2 mL of pancreatin solution (100 mg/mL), and 2 mL of bile salts (117 mg/mL). The final pH was adjusted to 6.5. Incubation was carried out at 41 °C and at the same 200 r/min for 0, 1, and 3 h. At each time point, digestion was stopped by flash-freezing in liquid nitrogen. Later on, the samples were thawed at 4 °C and centrifugation at 2,910 × *g* for 10 min (Beckman GS-6 centrifuge). Supernatants were collected and stored at −20 °C until nitrogen analysis, while the resulting pellets were lyophilized for structural assessment and determination of insoluble nitrogen. Samples from the 3 h intestinal phase were freeze-dried, hydrolyzed, and analyzed for amino acid composition as described by Njeri et al. [16]. In addition, in vivo AA standardized ileal digestibility (SID) data from a previous study conducted in our laboratory [2] using FFSB roasted at 136.7 °C were used to evaluate the predictive capability of the in vitro model with the current study sample, which was roasted at the same temperature. These in vivo samples were obtained from different batches and farms than those used in the present study; therefore, they were not intended for direct comparison but rather to assess the robustness and applicability of the in vitro prediction approach.

### Sample processing and laboratory analyses

Attenuated total reflectance Fourier-transform infrared (ATR-FTIR) spectroscopy was used to assess protein conformation on a Bruker Alpha II spectrometer equipped with a diamond crystal plate. A finely (1 mm) ground, lyophilized sample was applied to cover the crystal and compressed using the instrument’s pressure arm. Prior to each run, the crystal and anvil were cleaned with ethanol and dried. Spectra were collected over the range of 4,000–400 cm^−^^1^ at a resolution of 2 cm^−^^1^ (32 scans per sample). Spectral preprocessing, including baseline correction and smoothing (Savitzky–Golay, 9 points) was performed using OPUS software (Bruker Optics).

Secondary structure analysis focused on the Amide I region (1,600–1,700 cm^−^^1^), which was enhanced via Fourier self-deconvolution and curve-fitted using Voigt models in PeakFit v4.12 (SeaSolve Software Inc., USA). Peak assignments followed established guidelines: 1,600–1,610 cm^−^^1^ for sidechains, 1,610–1,620 cm^−^^1^ for aggregated antiparallel β-sheets, 1,620–1,640 cm^−^^1^ for β-sheets, 1,640–1,650 and 1,660–1,670 cm^−^^1^ for random coil, 1,650–1,660 cm^−^^1^ for α-helices, 1,670–1,680 cm^−^^1^ for antiparallel β-sheets, and 1,660–1,670/1,680–1,700 cm^−^^1^ for β-turns. Each sample was analyzed in triplicate, and structural parameters were averaged.

Protein profiles in the supernatant of undigested and digested samples were evaluated using sodium dodecyl sulfate-polyacrylamide gel electrophoresis (SDS-PAGE) under both reducing and non-reducing conditions as described by Njeri et al. [17]. The protein concentration of the undigested sample supernatants was determined using a bicinchoninic acid (BCA) assay kit. In brief, the samples were mixed with Laemmli buffer containing 2% SDS, 10% glycerol, 0.01% bromophenol blue, and 0.0625 mol/L Tris–HCl (pH 6.8). For reducing conditions, β-mercaptoethanol (or dithiothreitol) was added to the buffer, whereas it was omitted for non-reducing conditions. Samples were heated at 95 °C for 5 min prior to loading. Equal amounts of protein (10 µg per lane) were loaded onto polyacrylamide gels (12% resolving gel with 4% stacking gel). Electrophoresis was performed at a constant voltage (120 V) using a Mini-PROTEAN system (Bio-Rad Laboratories, Hercules, CA, USA) until the dye front reached the bottom of the gel. SDS-PAGE analysis was conducted using the Canadian reference samples, which represented the full range of defined processing conditions (known exit temperatures). This allowed for controlled comparison of protein profiles across treatments while maintaining consistent gel loading and interpretation.

Trypsin inhibitors were analyzed using the original method recommended by the American Oil Chemists' Society (AOCS) [18]. Dry matter (DM), soluble dietary fiber, insoluble dietary fiber, total dietary fiber, neutral detergent fiber, crude fat, AA were determined as done previously described by Njeri et al. [16, 17].

### Calculations and statistical analyses

In vitro crude protein (CP) solubilization was calculated from the nitrogen concentration remaining in the insoluble pellet at each digestion time point. Pellet nitrogen was converted to CP using a factor of 6.25, and CP solubilization was calculated as the proportion of initial sample CP that was no longer recovered in the insoluble pellet (Eq. 1).

1 $$\begin{aligned} &CP\ solubilization \left(\%\right)\\ &=\left[1-\frac{Pellet\ N\times 6.25}{Sample\ weight \times sample\ CP\ fraction}\right]\times 100 \end{aligned}$$

Protein secondary-structure variables and amino-acid digestibility were analyzed using the GLIMMIX procedure of SAS 9.4, with FFSB sample as a fixed effect and laboratory replicate as a random effect. Crude protein solubilization was analyzed separately within the gastric and intestinal phases, with FFSB sample, digestion time, and their interaction included as fixed effects and replicate included as a random effect. When the fixed effect of sample or the sample × time interaction was significant, least-squares means were separated using Tukey’s adjustment. Chemical-composition data were summarized descriptively because a single composite ingredient sample represented each source or processing condition. SDS-PAGE profiles and undeconvoluted ATR-FTIR spectra were interpreted qualitatively and were not statistically analyzed. Statistical significance was declared at *P* < 0.05.

Observed in vivo standardized ileal digestibility (SID) values were regressed against corresponding in vitro amino acid digestibility values for the 136.7 °C-roasted FFSB sample using PROC REG in SAS 9.4. Predicted values, residuals, absolute and squared prediction errors, 95% confidence limits for the mean, and 95% prediction intervals were generated from the fitted linear regression model (Eq. 2).

2 $${{Y}}_{\mathrm{InVivo}}=\beta_{0}+\beta_{1}{{X}}_{\mathrm{InVitro}}+\varepsilon$$

where:

- *Y*_InVivo_ = observed in vivo SID for each amino acid
- *X*_InVitro_ = corresponding in vitro digestibility at 136.7 °C

## Results

The analyzed chemical composition of raw, processed, and commercially sourced FFSB samples is presented in Table 1. Dry matter content varied within a narrow range across samples, differing by less than 2 percentage units. Roasted Canadian FFSB samples had slightly higher DM values (94.25%–94.85%) than raw (93.41%) and extruded FFSB (93.33%). The CP content ranged from 34.57% to 36.66% among Canadian FFSB samples and from 37.90% to 40.99% among Kenyan commercial samples. Within the Canadian samples, insoluble dietary fiber increased from 21.51% in raw FFSB to 23.31%–26.65% in roasted samples and 24.80% in extruded FFSB. Soluble dietary fiber was 1.36% in raw FFSB and ranged from 0.95% to 1.26% in processed Canadian samples. Crude fat content varied from 18.79% to 20.45% across samples, with no clear pattern related to origin or processing method. Trypsin inhibitor activity was highest in raw Canadian FFSB at 17.42 TIU/mg and was lower in processed Canadian samples, ranging from 2.30 to 4.77 TIU/mg in roasted samples and 3.85 TIU/mg in extruded FFSB. Kenyan commercial samples had TIA values ranging from 6.00 to 12.00 TIU/mg, which were lower than raw Canadian FFSB but higher than the processed Canadian samples. Amino acid composition showed minor variation among Canadian samples, whereas greater numerical variability was observed among Kenyan commercial samples.

**Fig. 1:**
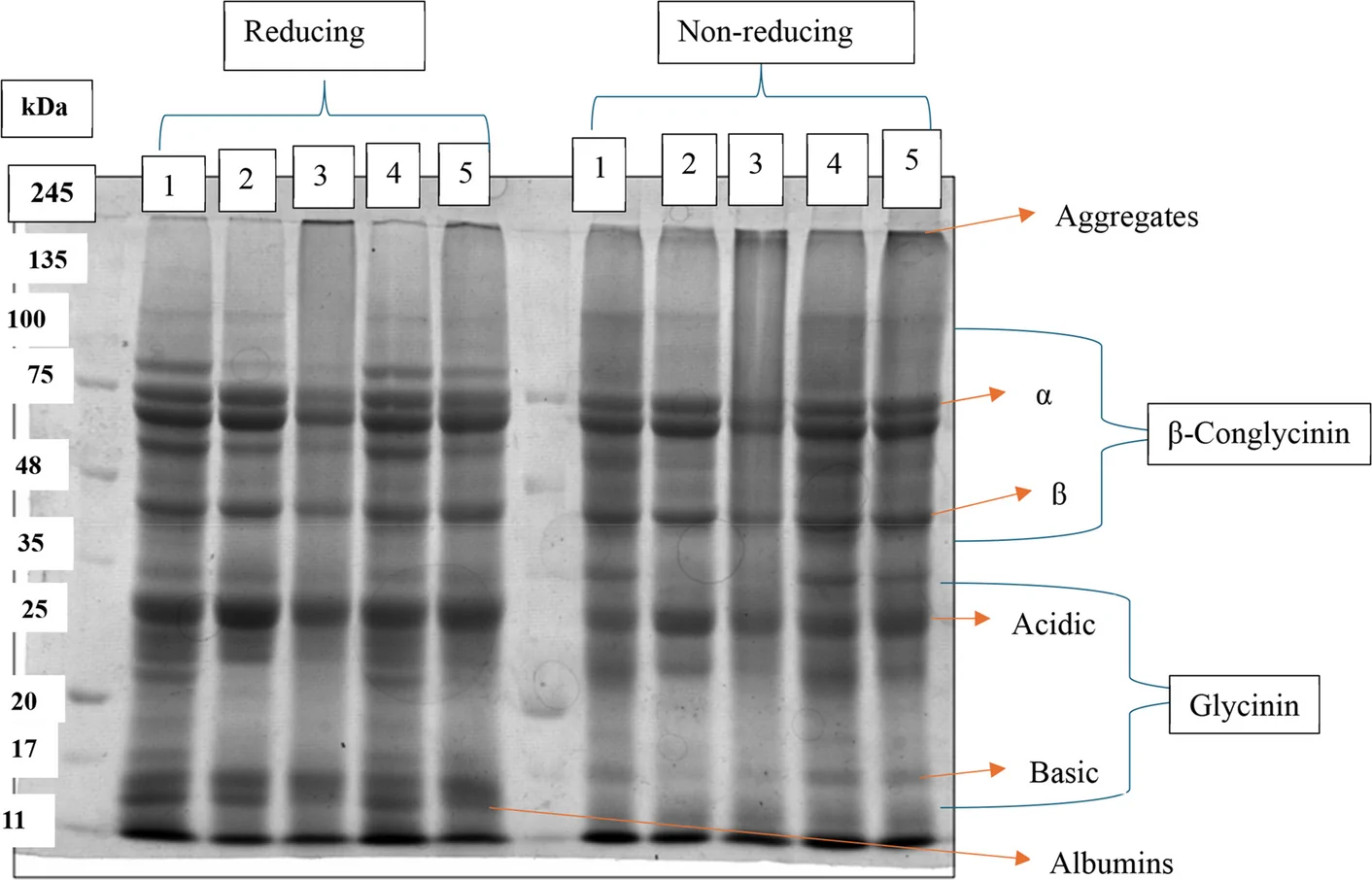
Sodium dodecyl sulfate-polyacrylamide gel electrophoresis (SDS-PAGE) profile of undigested protein from full-fat soybean (FFSB) processed under different heating conditions: (1) raw FFSB; (2) extruded FFSB (160 °C); (3) roasted FFSB (136.7 °C); (4) roasted FFSB (151.7 °C); and (5) roasted FFSB (154.4 °C). Molecular weight marker is shown in the leftmost lane

The SDS-PAGE profiles of undigested FFSB samples are shown in Fig. 1. Protein bands occurred at broadly similar apparent molecular-weight regions across samples, indicating retention of major protein fractions; however, band prominence varied visually under both reducing and non-reducing conditions. Under reducing conditions, raw FFSB showed distinct bands across the gel, while the Canadian sample roasted at 136.7 °C had weaker, less distinct bands than the extruded and higher-temperature roasted samples. Bands near 100 kDa were more prominent in samples roasted at 151.7 and 154.4 °C, and the 151.7 °C sample also showed clearer bands in the lower molecular-weight region (~11–25 kDa). Material retained near the top of the gel in samples roasted at 136.7 and 154.4 °C suggested high-molecular-weight aggregates or poorly resolved protein complexes. Under non-reducing conditions, bands were generally less distinct, consistent with reduced protein dissociation without reducing agent. Because band intensity was not quantified by densitometry, these observations are descriptive and were not statistically analyzed.

**Fig. 2:**
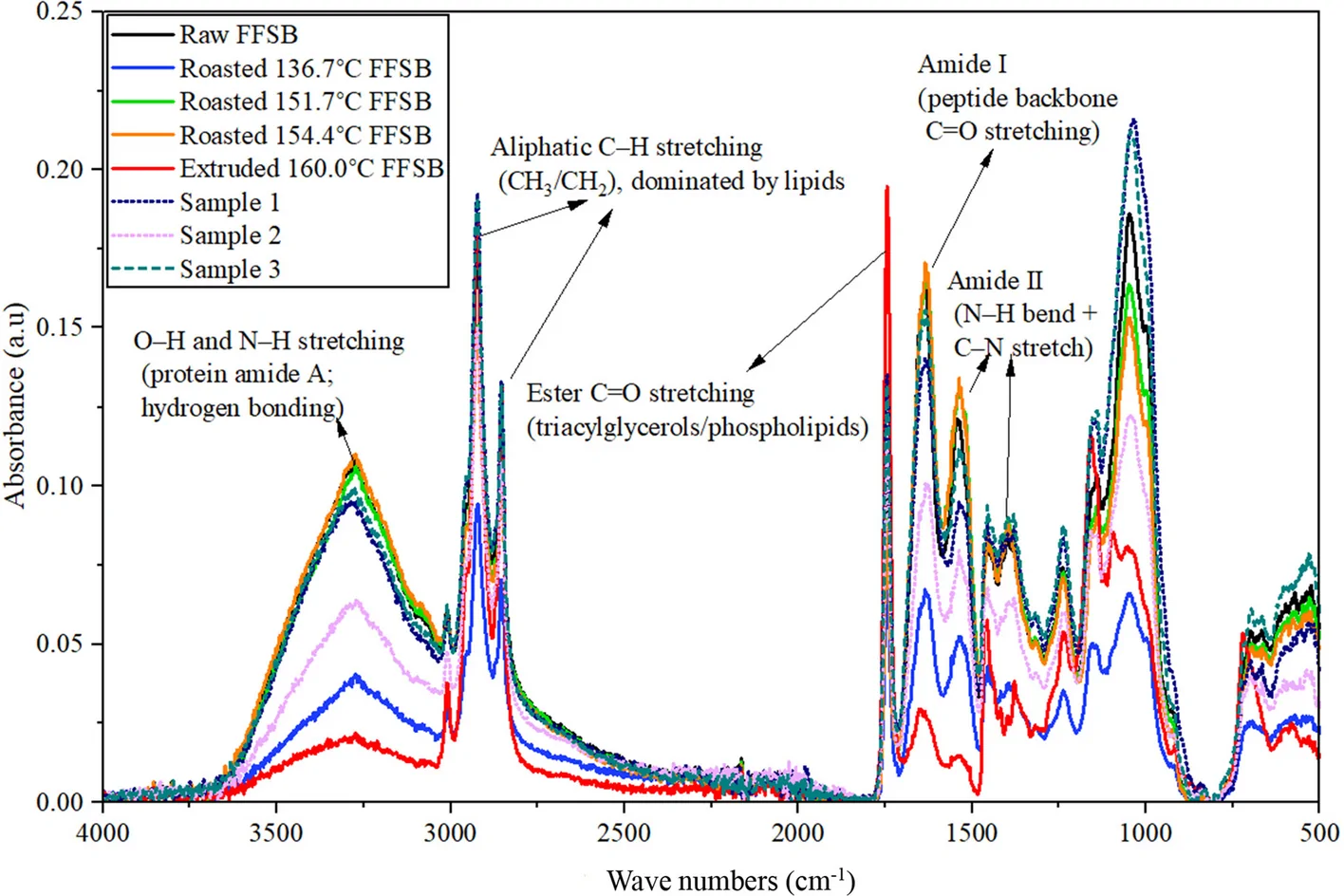
Attenuated total reflectance-Fourier transform infrared (ATR-FTIR) spectra of undigested full-fat soybean (FFSB) subjected to different processing conditions: (1) raw FFSB; (2) extruded FFSB (160 °C); (3) roasted FFSB (136.7 °C); (4) roasted FFSB (151.7 °C); and (5) roasted FFSB (154.4 °C)

The ATR-FTIR spectra of FFSB samples showed visual variation among samples across major functional group regions (Fig. 2). In the O–H and N–H stretching region, processed Canadian and Kenyan samples showed numerically reduced absorbance relative to raw FFSB, with the largest percentage reduction observed in extruded FFSB. In the aliphatic C–H stretching region, spectral profiles were broadly similar, with only small numerical differences in absorbance intensity.

**Fig. 3:**
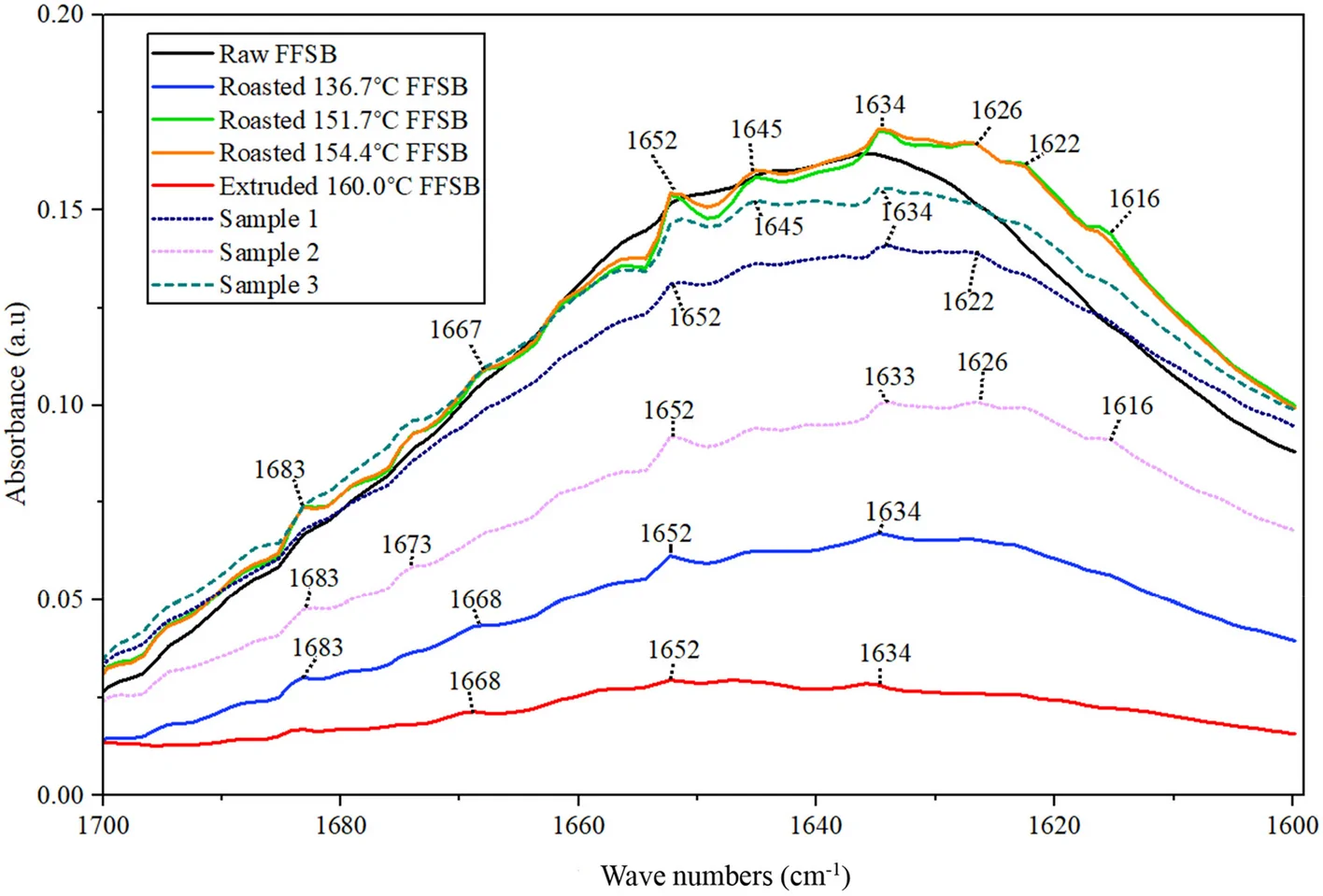
Expanded view of the Amide I region (1,700–1,600 cm^−^^1^) of undigested full-fat soybean (FFSB) under different processing conditions: (1) raw FFSB; (2) extruded FFSB (160 °C); (3) roasted FFSB (136.7 °C); (4) roasted FFSB (151.7 °C); and (5) roasted FFSB (154.4 °C)

**Fig. 4:**
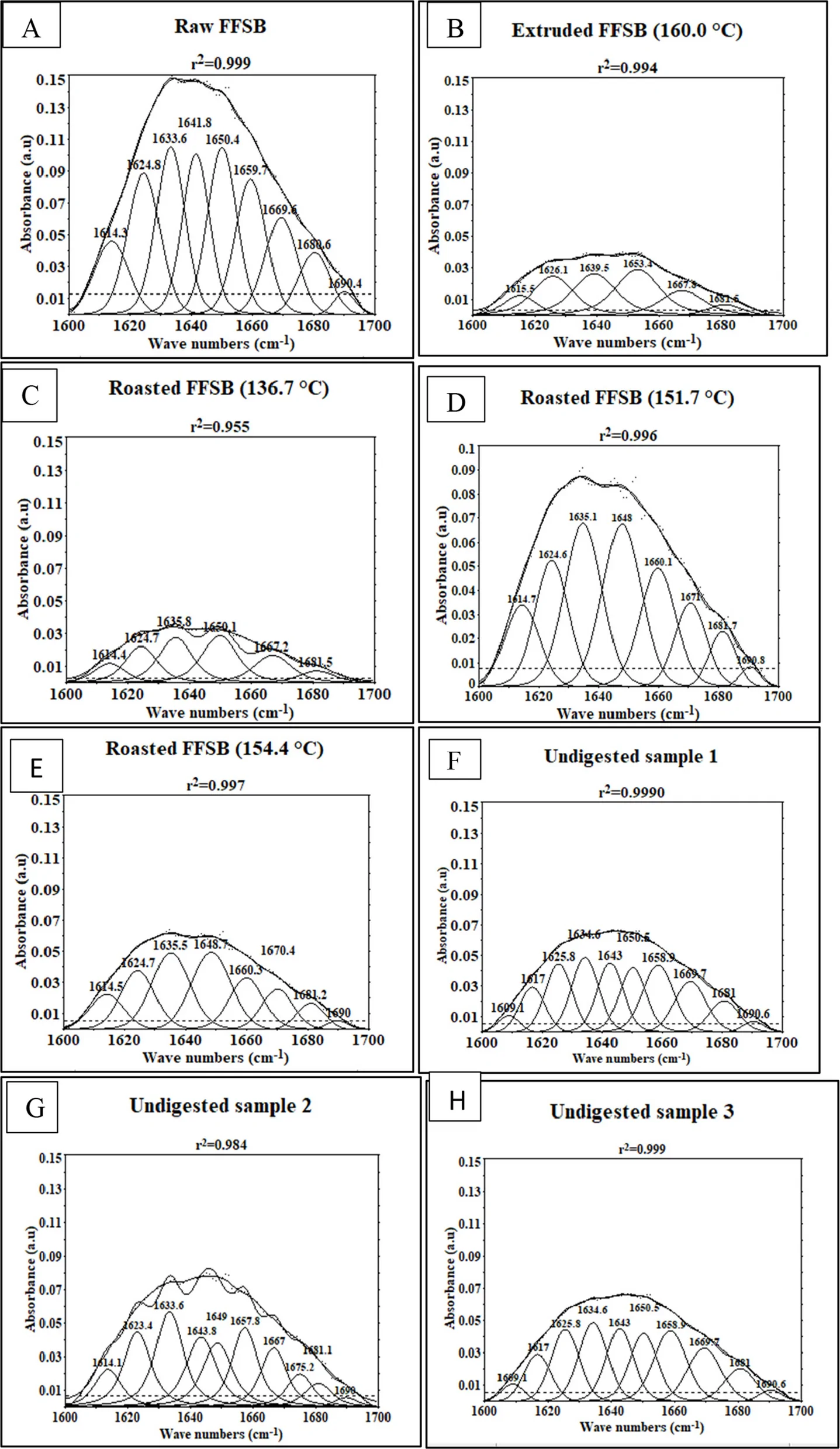
Deconvoluted ATR-FTIR spectrum of the Amide I region (1,700–1,600 cm^−^^1^) for undigested roasted full-fat soybean (FFSB) at raw (**A**), extruded at 160.0 °C (**B**), and roasted at temperatures of 136.7 °C (**C**), 151.7 °C (**D**), 154.4 °C (**E**), Sample 1(**F**), Sample 2 (**G**) and Sample 3 (**H**)

In the protein-associated regions, visual and numerical variation was observed in the Amide I and Amide II bands (Fig. 3). Compared with raw FFSB, the sample roasted at 136.7 °C and the extruded sample showed reduced Amide I/II absorbance and altered band profiles. The expanded (Fig. 3) and deconvoluted (Fig. 4) Amide I region showed changes in band shape and position, indicating processing-related alterations in protein secondary structure. Roasted samples above 136.7 °C showed distinct Amide I profiles relative to raw and extruded FFSB, whereas Kenyan samples showed greater numerical variability in absorbance distribution across the Amide I region.

Protein secondary structures of deconvoluted ATR-FTIR spectrum of the Amide I region (1,700–1,600 cm^−^^1^) composition differed among treatments (*P* < 0.001) (Table 2; Fig. 4). Roasting at 136.7 °C resulted in the highest aggregated β-sheet and β-turn contents and the lowest random coil content (*P* < 0.001). In contrast, random coil content was highest in Kenyan samples (Samples 1 and 3), followed by Canadian samples that were roasted at 151.7 °C and 154.4 °C. Intramolecular β-sheet was greatest in raw FFSB and roasted 151.7 °C, whereas lower values were observed in roasted 136.7 °C and Sample 1. α-Helix was highest in Kenyan Sample 2 and lowest in roasted 136.7 °C. Canadian extruded FFSB showed intermediate values for most structural components but had among the highest antiparallel β-sheet content, comparable to Kenyan Sample 3. Commercial samples sourced from the Kenyan market showed greater variability; Sample 2 had the highest α-helix and lowest aggregated β-sheet, whereas Samples 1 and 3 had the highest random coil contents.

**Table 2:** Secondary structure composition of intact full-fat soybean (FFSB) proteins determined by ATR-FTIR spectroscopy

Item, %^1^	Aggregated β-sheet	Intramolecular β-sheet	Random coil	α-Helix	β-Turn	Antiparallel β-sheet
Raw	5.604^de^	40.23^a^	19.80^c^	15.97^cb^	10.78^d^	7.614^ba^
Extruded at 160.0 °C	7.099^c^	38.04^b^	19.42^c^	16.43^b^	10.87^d^	8.141^a^
Roasted at 136.7 °C	9.374^a^	33.15^c^	14.45^d^	13.09^d^	24.14^a^	5.788^b^
Roasted at 151.7 °C	6.196^dc^	40.76^a^	22.01^b^	14.96^cb^	9.731^e^	6.346^ba^
Roasted at 154.4 °C	8.455^ba^	37.64^b^	22.66^b^	14.62^ cd^	9.570^e^	7.051^ba^
Sample 1	7.405BC	30.95^d^	28.05^a^	14.61^ cd^	11.61^d^	7.551^ba^
Sample 2	3.526^f^	34.68^c^	18.82^c^	19.43^a^	15.90^b^	7.640^ba^
Sample 3	4.669^fe^	33.26^c^	26.92^a^	14.39^ cd^	12.92^c^	7.838^a^
SEM	0.2468	0.3462	0.2621	0.3199	0.1897	0.3677
*P*-value	< 0.001	< 0.001	< 0.001	< 0.001	< 0.001	0.005

^a^^–^^d ^Means within a row with different superscripts differ significantly (*P* <0.05)

^1^Raw, extruded, and roasted samples represent Canadian reference full-fat soybean (FFSB) processed under defined conditions; roasting temperatures correspond to exit temperatures. Samples 1–3 were sourced from commercial vendors within the Kenyan market and represent FFSB with unknown processing histories

Crude protein solubilization differed among treatments at all time points during both gizzard and small intestinal (SI) phases (*P* < 0.001; Table 3). In the gizzard phase, at 0 h, raw FFSB showed the highest protein solubilization (*P* < 0.001), whereas extruded FFSB had the lowest, with roasted and commercial samples showing intermediate values. At 0.5 h, protein solubilization increased across all treatments, with raw FFSB remaining highest. Samples roasted at 151.7 and 154.4 °C showed higher solubilization than extruded FFSB and commercial Samples 1 and 3. In the intestinal phase, at 0 h, raw FFSB and Sample 2 had the highest solubilization, while Sample 1 and Sample 3 had the lowest values. At 1 h, roasted samples (136.7, 151.7, and 154.4 °C) and extruded FFSB showed higher solubilization than raw FFSB and Sample 2. At 3 h, roasted 154.4 °C had the highest solubilization, whereas raw FFSB and Sample 3 had the lowest values.

**Table 3:** The crude protein solubilization in full-fat soybean samples under in vitro simulated gastrointestinal digestion conditions

Time, h	Raw	Extruded 160.0 °C	Roasted 136.7 °C	Roasted 151.7 °C	Roasted 154.4 °C	Sample 1	Sample 2	Sample 3	SEM	*P*-value
Gizzard phase										
0	30.37^a^	8.58^d^	13.02^ cd^	15.30^c^	13.04^ cd^	9.66^ cd^	19.70^b^	13.20^ cd^	1.35	< 0.001
0.5	51.15^a^	38.65^c^	41.24^bc^	45.36^ba^	46.25^ba^	27.24^d^	42.80^bc^	28.14^d^	1.78	< 0.001
Small intestines phase										
0	66.14^a^	46.88^d^	52.44^c^	56.01^cb^	60.74^b^	39.83^e^	67.39^a^	42.07^e^	1.20	< 0.001
1	60.47^b^	68.72^ba^	76.51^a^	75.50^a^	77.17^a^	76.34^a^	60.57^b^	65.23^b^	2.44	< 0.001
3	59.30^c^	75.19^ba^	76.02^ba^	78.11^ba^	81.57^a^	75.46^b^	61.32^c^	60.49^c^	1.71	< 0.001

*SEM* Standard error of means

^a^^–^^e ^Means within a row with different superscripts differ significantly (*P* < 0.05)

**Fig. 5:**
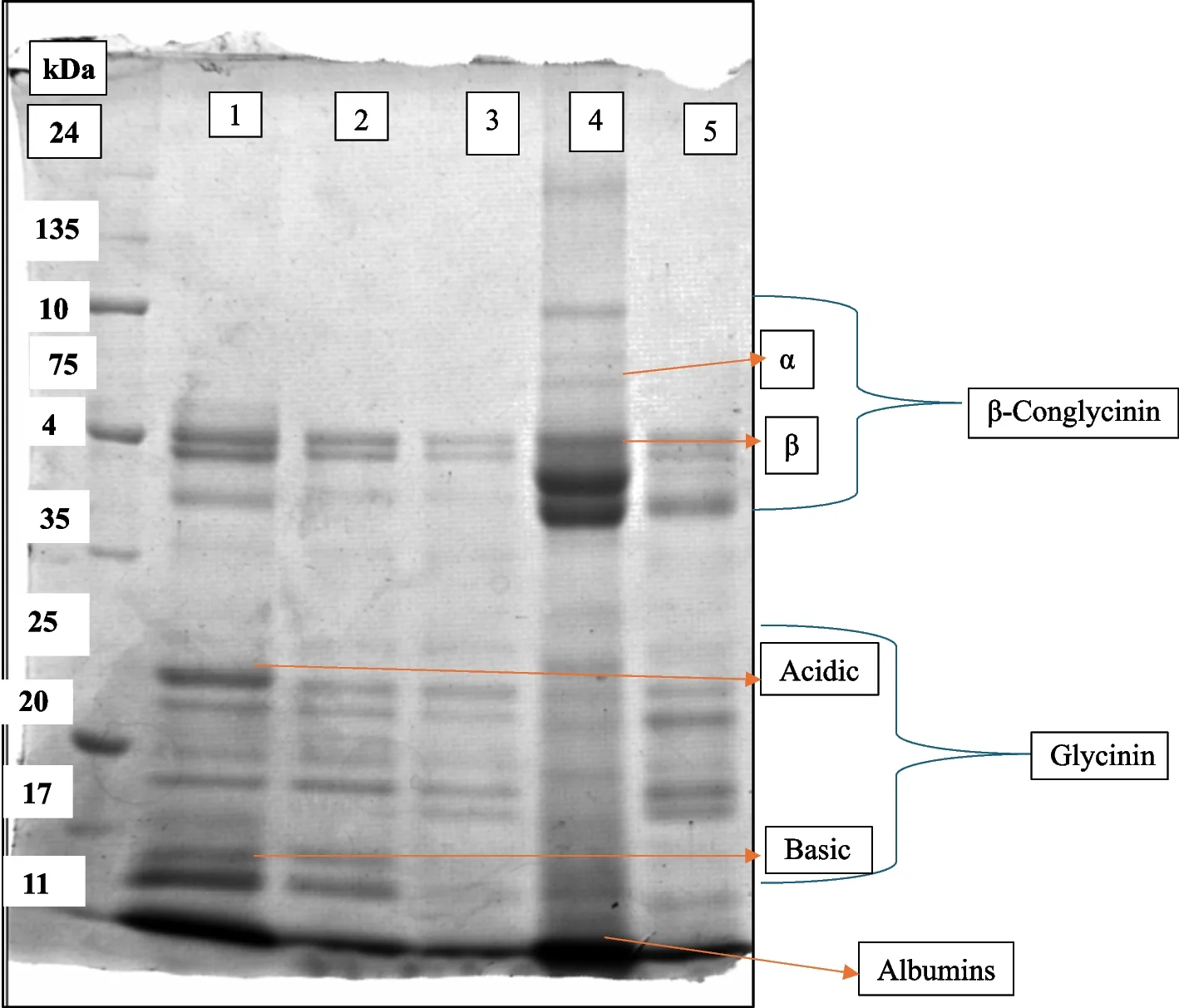
SDS-PAGE profile of proteins from full-fat soybean (FFSB) following in vitro digestion under simulated gastrointestinal conditions: (1) Raw FFSB; (2) Extruded FFSB (160 °C); (3) Roasted FFSB (136.7 °C); (4) Roasted FFSB (151.7 °C); and (5) Roasted FFSB (154.4 °C)

The SDS-PAGE profile of digested samples collected at the end of the small intestinal phase showed visual differences in molecular weight distribution among treatments (Fig. 5). High-molecular-weight bands at approximately 100 and 75 kDa were observed only in the sample roasted at 151.7 °C. A band near 48 kDa was present in all samples, with the greatest visual intensity observed in raw FFSB. At approximately 35 kDa, the band appeared most intense in the 151.7 °C sample, followed by the 154.4 °C sample, whereas this band was weak or not visible in the extruded and 136.7 °C-roasted samples. In the lower molecular weight region, multiple bands between approximately 11 and 25 kDa were observed across treatments. The 151.7 °C sample showed a lower visual band intensity in this region than the other processed samples. Among the processed samples, the extruded sample showed a relatively prominent band near 11 kDa, although it was less intense than in raw FFSB. The 151.7 °C sample also showed a distinct broad band below 11 kDa near the bottom of the gel.

In vitro amino acid digestibility differed among treatments for all indispensable and dispensable amino acids (*P* < 0.001; Table 4). Raw FFSB showed the lowest digestibility across amino acids. Thermal processing increased digestibility, with roasted samples generally showing higher values than extruded FFSB. Among experimental treatments, roasting at 154.4 °C consistently ranked among the highest, while roasting at 136.7 °C and extrusion showed intermediate responses. Commercial samples exhibited variability. Sample 1 showed the highest digestibility for most amino acids, whereas Sample 3 showed the lowest values, not different from raw FFSB for several amino acids. A similar pattern was observed for dispensable amino acids, and overall mean total amino acid digestibility followed the same treatment patterns.

**Table 4:** Effect of processing full-fat soybean samples on in vitro amino acid digestibility

Item, %^1^	Raw	Extruded 160.0 °C	Roasted 136.7 °C	Roasted 151.7 °C	Roasted 154.4 °C	Sample 1	Sample 2	Sample 3	SEM	*P*-value
Indispensable AA										
Arg	61.90^e^	79.50BC	80.07BC	81.51^b^	83.21^ba^	86.74^a^	75.53^c^	70.16^d^	1.04	< 0.001
His	56.82^e^	72.23^c^	74.72^cb^	76.41^cb^	79.70^b^	86.52^a^	76.82^cb^	63.58^d^	1.2	< 0.001
Ile	61.94^e^	76.99^c^	78.12^c^	79.81^cb^	82.96^b^	86.95^a^	78.55^c^	67.43^d^	0.674	< 0.001
Leu	59.55^e^	73.76^c^	76.48^cb^	78.01^b^	81.78^a^	84.90^a^	77.03^cb^	65.12^d^	0.687	< 0.001
Lys	69.52^c^	80.97^b^	80.36^b^	81.08^b^	83.94^a^	85.51^a^	78.51^b^	67.50^c^	0.53	< 0.001
Met	71.07^d^	71.43^ cd^	76.52^b^	75.98^cb^	81.56^a^	63.75^e^	76.31^cb^	66.05^e^	0.999	< 0.001
Phe	55.62^c^	89.65^a^	78.62^ba^	83.25^ba^	80.59^ba^	85.40^ba^	76.42^b^	64.95^c^	2.321	< 0.001
Thr	65.04^d^	72.32^c^	75.57^cb^	76.58^b^	80.72^a^	83.36^a^	73.79^cb^	58.58^e^	0.814	< 0.001
Val	64.78^e^	75.78^d^	76.78^d^	79.05BC	82.15^ba^	84.74^a^	76.04^dc^	63.24^e^	0.66	< 0.001
Mean Indispensable AA	62.92^d^	76.96^c^	77.47^c^	79.08BC	81.85^ba^	83.10^a^	76.55^c^	65.18^d^	0.748	< 0.001
Dispensable AA										
Ala	68.47^e^	75.91^ cd^	77.48^cb^	78.60^b^	82.57^a^	83.29^a^	74.40^d^	60.00^f^	0.446	< 0.001
Asp	67.57^d^	80.09^b^	80.14^b^	80.84^b^	84.51^a^	86.14^a^	76.83^c^	66.14^d^	0.509	< 0.001
Cys	86.04^a^	75.24^bac^	71.81BC	70.70BC	82.14^ba^	82.24^ba^	76.33^bac^	64.74^c^	2.609	0.001
Glu	67.99^d^	82.82^b^	81.77^cb^	82.58^b^	86.17^a^	87.84^a^	79.62^c^	70.44^d^	0.503	< 0.001
Gly	31.18^d^	55.32BC	60.63^bac^	62.90^ba^	65.78^a^	66.56^a^	52.03^c^	25.88^d^	1.992	< 0.001
Pro	62.90^c^	76.59^b^	77.39^b^	78.25^b^	81.93^a^	84.02^a^	75.38^b^	63.51^c^	0.691	< 0.001
Ser	57.24^d^	72.18^c^	75.05^c^	76.05BC	80.86^ba^	85.07^a^	74.62^c^	60.83^d^	1.009	< 0.001
Tyr	77.28^b^	83.43^ba^	83.77^ba^	82.84^ba^	82.61^ba^	69.94^c^	81.94^ba^	78.28^ba^	1.32	< 0.001
Mean dispensable AA	64.83^c^	75.20^b^	76.01^b^	76.59^b^	80.82^a^	80.64^a^	73.90^b^	61.23^c^	0.768	< 0.001
Mean total AA	63.82^d^	76.13^c^	76.78^c^	77.91BC	81.36^ba^	81.94^a^	75.30^c^	63.32^d^	0.732	< 0.001

Values are means of triplicate observations expressed as percentage digestibility

^a^^–^^e ^Means within a row with different superscripts differ significantly (*P* < 0.05)

*SEM* Standard error of the mean

^1^Raw, extruded, and roasted samples represent Canadian reference full-fat soybean (FFSB) processed under defined conditions; roasting temperatures correspond to exit temperatures. Samples 1–3 were sourced from commercial vendors within the Kenyan market and represent FFSB with unknown processing histories. Amino acid digestibility values were determined using an in vitro digestion model simulating gastric and intestinal phases

Predicted standardized ileal digestibility (SID) values were generally close to observed in vivo values, with residuals ranging from −6.58 to 6.11 percentage units (Table 5). Prediction accuracy differed among amino acids. Larger absolute errors were observed for aspartic acid, proline, and lysine, whereas histidine, isoleucine, and valine showed smaller errors. Confidence intervals for mean predicted values (LCLM–UCLM) were relatively narrow, whereas prediction intervals (LCL_PI–UCL_PI) were wider across amino acids. Residuals included both positive and negative values across amino acids.

**Table 5:** Observed and model-derived predicted standardized ileal digestibility of amino acids in full-fat soybean samples digested in vitro at 136.7 °C, with amino-acid-specific prediction error and uncertainty intervals

Item, %	*Y*_InVivo_^1^	*X*_InVitro_^2^	Predicted^3^	Resid^4^	AbsErr^4^	SqErr^4^	LCLM^5^	UCLM^5^	LCL_PI^6^	UCL_PI^6^
Arg	86.90	80.07	83.18	3.72	3.72	13.83	78.13	88.23	73.08	93.28
His	85.20	74.72	85.30	−0.10	0.10	0.01	80.05	90.56	75.10	95.51
Ile	83.10	78.13	83.95	−0.85	0.85	0.72	80.86	87.05	74.67	93.23
Leu	84.20	76.48	84.61	−0.41	0.41	0.16	81.29	87.92	75.25	93.96
Lys	77.10	80.36	83.07	−5.97	5.97	35.60	77.64	88.50	72.77	93.36
Met	88.90	76.52	84.59	4.31	4.31	18.60	81.31	87.87	75.24	93.93
Phe	87.20	78.62	83.75	3.45	3.45	11.88	80.31	87.20	74.35	93.16
Thr	81.10	75.57	84.96	−3.86	3.86	14.94	80.77	89.16	75.26	94.67
Val	84.20	76.78	84.49	−0.29	0.29	0.08	81.37	87.60	75.20	93.77
Ala	83.30	77.48	80.73	2.57	2.57	6.60	76.88	84.58	69.58	91.89
Asp	75.30	80.14	81.88	−6.58	6.58	43.31	77.82	85.94	70.65	93.11
Cys	74.80	71.81	78.28	−3.48	3.48	12.10	70.49	86.07	65.23	91.33
Glu	81.30	81.77	82.59	−1.29	1.29	1.65	77.60	87.57	70.99	94.18
Gly	80.90	80.63	82.10	−1.20	1.20	1.43	77.80	86.39	70.78	93.41
Pro	86.80	77.39	80.69	6.11	6.11	37.28	76.82	84.57	69.53	91.86
Ser	81.60	75.05	79.68	1.92	1.92	3.69	74.56	84.80	68.02	91.33
Tyr	85.40	83.78	83.45	1.95	1.95	3.79	76.91	89.99	71.11	95.80

^1^In vivo values represent standardized ileal digestibility (SID) of individual amino acids [2]

^2^In vitro digestibility was measured at 136.7 °C and represents the mean of triplicate determinations for each amino acid

^3^Predicted values were obtained from a single linear regression model relating in-vitro digestibility at 136.7 °C to in-vivo standardized ileal digestibility

^4^Resid denotes the signed difference between observed and predicted in-vivo digestibility (Observed − Predicted); AbsErr denotes the absolute prediction error; SqErr denotes the squared prediction error

^5^LCLM and UCLM represent the lower and upper 95% confidence limits for the mean predicted value

^6^LCL_PI and UCL_PI represent the lower and upper 95% prediction limits for individual amino acids, reflecting uncertainty in individual-level prediction

Wide prediction intervals indicate limited precision of in-vitro digestibility for predicting individual amino acid SID digestibility, particularly for amino acids with distinct metabolic or chemical characteristics

## Discussion

The present study demonstrated that the nutritional quality of FFSB was determined by the combined effects of processing history, residual trypsin inhibitor activity (TIA), protein structural modification, and digestive behavior rather than by heating temperature alone. The Canadian reference samples processed under defined conditions and the commercially sourced Kenyan samples differed substantially in protein secondary structure, CP solubilization, and in vitro amino acid (AA) digestibility. These findings support the concept of an optimal processing window in which sufficient thermal input is required to inactivate antinutritional factors and increase enzyme accessibility, while excessive or poorly controlled processing may promote aggregation, cross-linking, or AA damage [19]. Accordingly, exit temperature should not be interpreted independently of moisture, residence time, heat-transfer efficiency, equipment design, and mechanical shear.

Residual TIA remained a useful indicator of processing adequacy but did not independently explain protein digestion. Raw Canadian FFSB had the highest TIA at 17.42 TIU/mg, whereas extrusion and roasting reduced TIA to 2.30–4.77 TIU/mg. Kenyan commercial samples retained 6.00–12.00 TIU/mg, indicating less complete or less consistent processing. These values were biologically relevant because increasing soybean TIA has been associated with reduced AA digestibility, pancreatic enlargement, lower growth, and poorer feed efficiency in broilers [20–23]. However, the contrasting digestive responses among samples indicate that TIA should be interpreted together with processing history and protein structure.

The three Kenyan samples showed distinct combinations of TIA, structural characteristics, and digestibility. Sample 1 had elevated TIA but the greatest or among the greatest digestibility values for most AAs, together with high random-coil and relatively low intramolecular β-sheet contents. This diverges from the generally negative relationship between TIA and AA utilization reported in broilers [20–23], and suggests that its comparatively unfolded protein structure may have contributed to greater enzyme accessibility, although the independent effects of TIA and structure were not experimentally separated. Sample 2 had the lowest TIA, the highest α-helix content, the lowest aggregated β-sheet content, and intermediate digestibility. Its high initial intestinal crude protein (CP) solubilization was not sustained at later time points, supporting evidence that solubility and proteolytic digestion are related but distinct properties influenced by matrix structure and enzyme accessibility [19, 24]. Sample 3 had the highest TIA and the lowest or among the lowest intestinal CP solubilization and AA digestibility, consistent with inadequate or inconsistent processing. Although it originated from a production context reportedly associated with elevated mortality, no health, diet, disease, management, or environmental data were available; therefore, no conclusions regarding mortality can be drawn.

Raw Canadian FFSB showed high gastric CP solubilization but low intestinal solubilization and AA digestibility. Its initial solubilization may reflect acid-phase extraction of native soybean proteins, whereas residual TIA and compact native structure likely constrained subsequent pancreatic digestion. Kakade et al. [25] demonstrated that removal of trypsin inhibitors from unheated soybean protein improved protein digestibility, while Gilani et al. [26] reviewed the adverse effects of protease inhibitors on protein digestion and AA availability. The present findings extend those reports by showing that high gastric solubilization did not translate into high intestinal digestion. Therefore, early acid-phase solubilization should not be interpreted as evidence of high nutritional value.

Extrusion substantially reduced TIA but produced only intermediate AA digestibility. The extruded sample also had the lowest initial gastric solubilization and relatively high antiparallel β-sheet content. Chen et al. [27] demonstrated that extrusion can induce soybean protein cross-linking and aggregation, particularly under low-moisture conditions. Thus, effective inhibitor inactivation may have been partly counterbalanced by structural changes that reduced enzyme accessibility. This agrees with broiler studies showing that extruded soybean products can support high digestibility when adequately processed, but their feeding value depends on the combined effects of temperature, moisture, pressure, and mechanical shear [2, 28].

The 136.7 °C-roasted sample had the highest aggregated β-sheet and β-turn contents and the lowest random-coil and α-helix contents. Its AA digestibility was greater than that of raw FFSB but lower than that of samples roasted at 151.7 and 154.4 °C. This agrees with Zhang et al. [19], who associated low-moisture heating with β-sheet-rich aggregation and reduced digestion, and with Duque-Estrada et al. [24], who showed that digestion of processed soy matrices was influenced by matrix structure and enzyme-accessible surface area. Because the 136.7 °C sample had a longer residence time than the 154.4 °C sample, its response likely reflected total thermal history rather than exit temperature alone; it may also have been extruded rather than roasted.

The most favorable responses occurred in samples roasted at 151.7 °C and 154.4 °C. Both had low TIA, high intestinal CP solubilization, and high mean indispensable, dispensable, and total AA digestibility. Their greater random-coil content was consistent with increased unfolding and enzyme accessibility. Similar improvements in soybean protein digestion after appropriate combinations of heat and moisture were reported by Zhang et al. [19]. In broilers, Thanabalan et al. [2] reported high standardized ileal digestibility (SID) for adequately processed FFSB, while Abdollahi et al. [29] showed that wet heating, expansion temperature, and autoclaving duration affected FFSB energy and AA value. However, the similarity between the 151.7 °C and 154.4 °C treatments does not establish a universally optimal temperature because processing duration, moisture, and heat-transfer conditions differed. Both inadequate and excessive processing can impair AA digestibility and broiler performance [29, 30].

Although random-coil content generally coincided with higher digestibility in several samples, this relationship was not consistent across all treatments. Zhang et al. [19] and Duque-Estrada et al. [24] similarly demonstrated that soybean protein digestion depends on multiple interacting characteristics, including oxidation, β-sheet formation, aggregation, matrix structure, and enzyme contact. Therefore, ATR-FTIR measurements should be interpreted as complementary indicators of processing-related structural changes rather than independent predictors of nutritional value.

The SDS-PAGE profiles were consistent with treatment-dependent changes in protein association and hydrolysis. Differences in band definition and material retained near the gel origin resembled heat-induced high-molecular-weight assemblies reported in soybean protein systems [19, 31]. After intestinal digestion, visible high- and intermediate-molecular-weight bands remained in the 151.7 °C sample despite its high CP solubilization and AA digestibility. These bands may represent soluble or partially hydrolyzed fragments rather than indigestible proteins, while smaller peptides may have migrated beyond the resolving range. Because densitometry and bond-specific analyses were not performed, the gel profiles should be interpreted qualitatively and together with CP solubilization and AA disappearance.

Thermal processing improved the digestibility of most indispensable AAs, although responses differed among individual AAs. The 151.7 and 154.4 °C samples had high digestibility of most indispensable AAs, consistent with broiler studies showing improved SID after adequate FFSB processing [2, 29]. The practical relevance of these responses is supported by studies in which reduced AA digestibility in improperly processed soybean products was accompanied by poorer growth or feed efficiency [30]. Thus, the high in vitro digestibility of the two higher-temperature roasted samples indicates favorable nutritional potential, although direct feeding validation is required.

Lysine remained highly digestible in the 151.7 and 154.4 °C samples, suggesting that these treatments did not cause severe heat damage. Nevertheless, lysine had one of the largest in vitro–in vivo prediction errors. Fontaine et al. [32] demonstrated that heat processing may reduce reactive lysine even when total lysine does not fully indicate the damage, while Parsons et al. [33] reported lower lysine availability in overprocessed soybean meal. The prediction error may therefore reflect differences in reactive lysine, limitations of the in vitro assay, or the use of unmatched in vitro and in vivo ingredient batches.

Methionine and cysteine showed treatment patterns that differed from those of most other AA. The 154.4 °C sample had high methionine digestibility, whereas Sample 1 had a comparatively low value despite its high mean indispensable AA digestibility. Cysteine digestibility was numerically high in raw FFSB but lower or intermediate in several processed samples. Fontaine et al. [32] and Parsons et al. [33] reported that heating altered the release or availability of sulfur-containing AAs in soybean proteins. Oxidation, disulfide rearrangement, and cross-linking could therefore have contributed to the present responses. However, sulfur-AA oxidation products were not measured, and cysteine had a relatively large SEM; consequently, these mechanisms remain speculative.

The response of dispensable AA generally followed that of indispensable AAs. The 154.4 °C sample and Sample 1 had high digestibility of most dispensable AAs, whereas raw FFSB and Sample 3 had low values for several AAs, particularly glycine. Because the in vitro assay contained no intestinal mucosa or endogenous protein secretions, these differences reflect variation in the enzymatic release or recovery of ingredient-derived AAs rather than endogenous losses. Boisen and Fernández [34] also reported variable prediction accuracy among individual dispensable AAs, including proline and glutamic acid.

The exploratory regression showed reasonable agreement at the mean level, but prediction accuracy varied among individual AAs, with the greatest errors for aspartic acid, proline, and lysine. This agrees with Boisen and Fernández [34] and Święch [35], who showed that static enzymatic methods may predict overall protein digestion more reliably than every individual AA. Unlike the in vitro assay, in vivo SID is influenced by gastrointestinal transit, digestive secretions, endogenous AA losses, absorption, microbial activity, and physiological regulation [36, 37]. Precision was further limited because the model used one processing condition and unmatched in vitro and in vivo batches. The model should therefore be considered exploratory, and future validation should use matched batches, multiple processing conditions, and independent datasets.

The Kenyan and Canadian samples differed in composition, TIA, secondary structure, and digestive responses. Similar variation in composition, metabolizable energy, and SID has been reported among soybean meals from different producing regions [38]. However, geographic sources were confounded with genotype, agronomic conditions, storage, post-harvest handling, and processing history. The observed differences therefore represent variability among distinct production and processing systems rather than an independently demonstrated effect of geographic origin.

## Conclusion

Overall, FFSB quality assessment should not rely on TIA, processing temperature, or a single structural index alone. Raw FFSB and Sample 3 showed unfavorable digestive profiles, while the extruded and 136.7 °C-roasted samples had reduced TIA but only intermediate digestibility. In contrast, roasting at 151.7 and 154.4 °C produced the most favorable combination of low TIA, protein structural modification, intestinal CP solubilization, and AA digestibility under the evaluated conditions. These findings agree with animal studies showing that adequately processed FFSB supports favorable AA utilization, whereas inadequate or excessive processing impairs digestibility, growth, or efficiency. Consistent production of high-quality FFSB therefore requires control of the complete thermal process, including temperature, moisture, residence time, mechanical energy, and heat-transfer efficiency.

## Supplementary Information

Supplementary Material 1. Full uncropped gel images.

## Data Availability

The datasets generated and analyzed during the current study are available from the corresponding author upon reasonable request.

## Abbreviations

AA: Amino acid
ADF: Acid detergent fiber
ANF: Antinutritional factor
ATR-FTIR: Attenuated total reflectance-Fourier transform infrared spectroscopy
CP: Crude protein
DM: Dry matter
FFSB: Full-fat soybean
IDF: Insoluble dietary fiber
NDF: Neutral detergent fiber
SDS-PAGE: Sodium dodecyl sulfate-polyacrylamide gel electrophoresis
SID: Standardized ileal digestibility
SDF: Soluble dietary fiber
TIA: Trypsin inhibitor activity
