# Supplementary material for: Full-fat soybean origin and thermal processing affect protein secondary structure, in vitro digestion kinetics, and predicted standardized ileal amino acid digestibility in broiler chickens
Source: J Anim Sci Biotechnol. 2026 Nov 2;17:197. doi: 10.1186/s40104-026-01512-w (PMC13726081; doi:10.1186/s40104-026-01512-w)
Supplement: Supplementary file 1 [file 40104_2026_1512_MOESM1_ESM.docx]

**Fig. S1** Full uncropped gel corresponding to Figure 1 in the manuscript. Lane M = molecular weight marker; Lane 1 = Raw FFSB; Lane 2 = Extruded FFSB at 160.0 °C; Lane 3 = Roasted FFSB at 136.7 °C; Lane 4 = Roasted FFSB at 151.7 °C; Lane 5 = Roasted FFSB at 154.4 °C

5

4

2

2

1

5

4

3

2

1

**245**

**135**

**100**

**75**

**48**

**35**

**25**

**20**

**17**

**11**

M

M

Non-reducing

Reducing

1

2

3

4

5

2

1

8

7

61

**245**

**135**

**100**

**75**

**48**

**35**

**25**

**20**

**17**

**11**

M

M

**Fig. S2** Full uncropped gel corresponding to Figure 5 in the manuscript. Lane M = molecular weight marker; Lane 1 = Raw FFSB; Lane 2 = Extruded FFSB at 160.0 °C; Lane 3 = Roasted FFSB at 136.7 °C; Lane 4 = Roasted FFSB at 151.7 °C; Lane 5 = Roasted FFSB at 154.4 °C; Lane 6 = Kenyan FFSB S1; Lane 7 = Kenyan FFSB S2; Lane 8 = Kenyan FFSB S3. The boxed area indicates the cropped region shown in the manuscript
